# An Update on Cholera Immunity and Current and Future Cholera Vaccines

**DOI:** 10.3390/tropicalmed6020064

**Published:** 2021-04-28

**Authors:** Jan Holmgren

**Affiliations:** University of Gothenburg Vaccine Research Institute, Sahlgrenska Academy, University of Gothenburg, 40530 Gothenburg, Sweden; jan.holmgren@gu.se

**Keywords:** cholera, oral cholera vaccine, mucosal immunity, cholera control

## Abstract

Individual resistance to cholera infection and disease depends on both innate host factors and adaptive immunity acquired by a previous infection or vaccination. Locally produced, intestinal-mucosal secretory IgA (SIgA) antibodies against bacterial surface lipopolysaccharide (LPS) O antigens and/or secreted cholera toxins are responsible for the protective adaptive immunity, in conjunction with an effective mucosal immunologic memory that can elicit a rapid anamnestic SIgA antibody response upon re-exposure to the antigen/pathogen even many years later. Oral cholera vaccines (OCVs), based on inactivated *Vibrio cholerae* whole-cell components, either together with the cholera toxin B subunit (Dukoral™) or administered alone (Shanchol™/Euvichol-Plus™) were shown to be consistently safe and effective in large field trials in all settings. These OCVs are recommended by the World Health Organisation (WHO) for the control of both endemic cholera and epidemic cholera outbreaks. OCVs are now a cornerstone in WHO’s global strategy found in “Ending Cholera: A Global Roadmap to 2030.” However, the forecasted global demands for OCV, estimated by the Global Alliance for Vaccines and Immunization (GAVI) to 1.5 billion doses for the period 2020–2029, markedly exceed the existing manufacturing capacity. This calls for an increased production capacity of existing OCVs, as well as the rapid introduction of additional and improved vaccines under development.

## 1. Introduction

Large placebo-controlled field trials in different parts of the world in the 1960s revealed that the parenteral inactivated whole-cell cholera vaccines, which have been used widely since the early 1900s, gave little protection. This led the World Health Organisation (WHO) to remove its recommendations and most countries to abandon cholera vaccination in the 1970s [1]. 

It then took until 2010 before WHO was again recommending countries to use cholera vaccination in the public health control of both endemic and epidemic cholera, which was now based on oral cholera vaccines (OCVs) with much greater protective effectiveness and acceptability than the abandoned parenteral vaccines [2]. OCVs are now a cornerstone in the action plan “Ending Cholera: A Global Roadmap to 2030,” which was launched in 2017 by WHO’s Global Task Force on Cholera Control (GTFCC), together with 50 additional organizations. The goals are to reduce cholera deaths by at least 90% and eliminate cholera transmission in most of the currently afflicted countries by 2030 [3]. 

**Figure 1:**
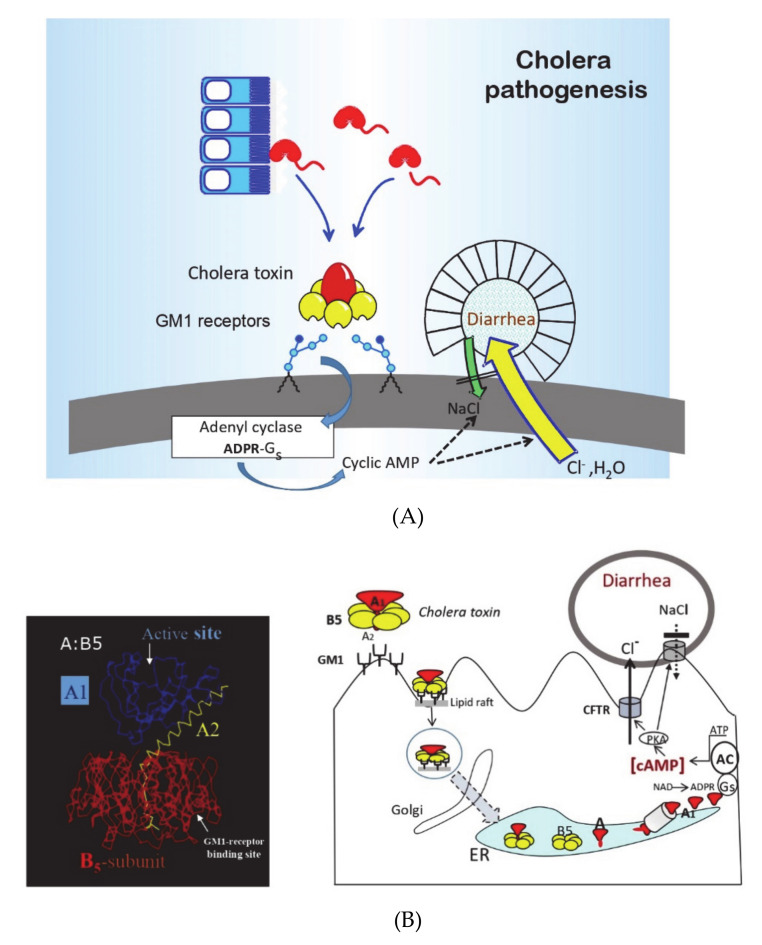
Pathogenesis of cholera and mode of action of the cholera toxin. (**A**) In the 1970s, the pathogenesis of cholera rapidly became better understood than any other infectious disease, as summarized in this figure from a Nobel Symposium on cholera in 1978 (A-M. Svennerholm, p.162 in [4]). After ingesting contaminated food or water, *V. cholerae* bacteria colonize the small intestine and secrete the cholera toxin, a doughnut-like protein with a central enzymatic toxic-active A (A_1_ + A_2_) subunit that is associated with a cell-binding pentamer of B subunits (B5). After binding to cell surface receptors identified as the GM1 ganglioside (the first-ever structurally defined mammalian cell receptors), the A subunit dissociates from the B subunits and its A1 entity binds to and ADP-ribosylates the GTP-binding Gs adenyl cyclase protein. This leads to the production of cyclic AMP (cAMP), which in turn induces the secretion of chloride, bicarbonate, and water from intestinal crypt cells and blocks sodium chloride and water uptake from villus cells, resulting in the watery diarrhea, dehydration, and acidosis that is typical of severe cholera. (**B**) Subsequent crystallographic studies have confirmed the A:B5 dough-nut structure of the cholera toxin and further detailed knowledge has been gained about the way the cholera toxin induces fluid secretion. After binding to GM1 ganglioside receptors, which are mainly localized in lipid rafts on the cell surface, the toxin is endocytosed and, via a retrograde pathway, travels to the endoplasmic reticulum (ER). In the ER, the A subunit dissociates from the B subunits and, through translocation via the ER degradosome pathway, A1 is released into the cytosol. After refolding, A1 ADP-ribosylates Gs, stimulating the adenyl cyclase (AC) complex to produce increased levels of cAMP, leading to the activation of protein kinase A (PKA), phosphorylation of the major chloride channel CFTR (the cystic fibrosis transmembrane conductance regulator), and the secretion of chloride (Cl^−^), among other effects, resulting in the often lethal cholera diarrhea and fluid loss.

**Figure 2:**
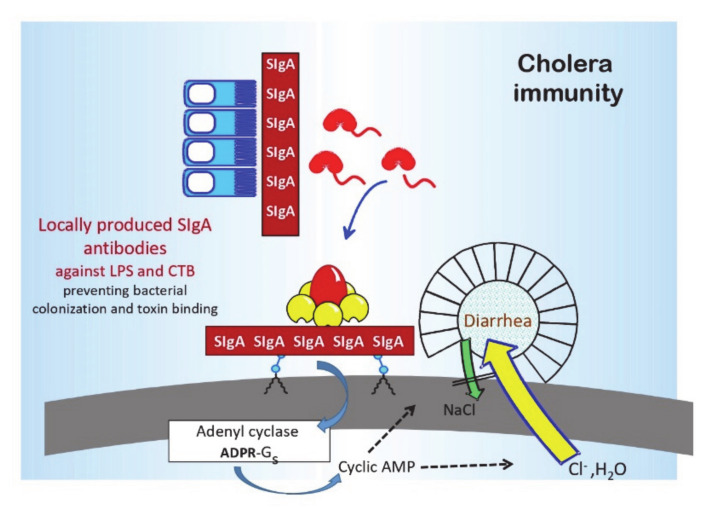
Protective immunity in cholera. Immune protection after infection or oral immunization is mediated mainly, if not exclusively, by locally produced SIgA antibodies that are directed against the cell surface LPS O antigen (predominantly against the A epitope defining the O1 serogroup, but also against the serotype-specific epitopes B (Ogawa) and C (Inaba) and the cholera toxin (mainly against the B subunit pentamer), and which inhibit bacterial colonization and toxin binding, respectively.

The development of the first effective OCV, namely, Dukoral™ was a result of an exceptionally successful era of international cholera research in response to the seventh cholera pandemic that began in the 1960s. Before that, most cholera research had been restricted to India. However, the rapid spread of cholera throughout Southeast Asia in the 1960s and into and across Africa in the 1970s attracted a wide range of scientists internationally. Geopolitical and military considerations of the time also mobilized increased funding to cholera research, especially in the USA and Japan. As summarized in a Nobel Symposium on cholera and related diarrheas in 1978 [4], in a “golden” research decade in the 1960s and 1970s, the pathophysiology, pathogenesis, and immune mechanisms of cholera had become better defined than any other infectious disease (see Figure 1 and Figure 2, [5,6]). Cholera was recognized as the archetype and the “tip of the iceberg” of a whole new entity of “enterotoxic enteropathies,” with enterotoxigenic *E. coli* (ETEC) as the most important additional pathogen. Practically, as had been discussed previously, the discovery and clinical introduction of life-saving oral rehydration therapy (ORT) had dramatically improved the clinical management of cholera and other diarrheal diseases, and as described below, the new knowledge about cholera pathogenesis and immunity had paved the way for the development of new, effective OCVs.

## 2. History of Vaccine Development 

The development of cholera vaccines began almost immediately after the rediscovery and culture of *Vibrio comma (cholerae)* as the causative agent of cholera by Robert Koch in 1884 (the original discovery by Filipo Pacini in Italy in 1854 was essentially long forgotten until the international committee on nomenclature in 1965 adopted *Vibrio cholerae Pacini 1854* as the correct name of the cholera-causing organism). As reviewed by Lopez et al. [1], Ferran in Spain in the same year produced a killed bacterial vaccine, which he gave parenterally to thousands of people in an area experiencing a cholera epidemic at the time. Of those vaccinated, 1.3% got cholera compared with 7.7% of those not vaccinated. Shortly thereafter, in India, Haffkine gave a similar parenteral killed cholera vaccine to people in cholera-afflicted slums in Delhi and Calcutta (Kolkata) and noted a marked protective effect against cholera deaths. In the 1920s, Russell reported an approximately 80% protective efficacy during a 3-month follow-up period after large-scale vaccination trials in India. As a result, the parenteral cholera vaccine became widely used in Southeast Asia, especially in expatriates. In fear of cholera epidemics and with recommendations from the WHO, many countries also required cholera vaccination certificates for the entry of travelers. 

However, as mentioned, several controlled studies from East Pakistan (now Bangladesh), India, the Philippines, and Indonesia during the 1960s showed that cholera vaccination gave only modest protection (approximately 50% for only a few months) and was limited to adults. Some vaccine preparations had apparent higher efficacy but were also associated with higher rates of adverse reactions, such as fever and local pain and swelling. Based on these results, in the 1970s, the WHO withdrew its previous recommendations for killed parenteral cholera vaccines.

The interest instead progressively turned to the development of orally administered cholera vaccines. The oral vaccination concept was not new. In the 1920s, a killed whole-cell OCV given together with ox bile (which had been found by Besredka to increase the immunogenicity of his killed oral *Shigella dysenterie* vaccine) was tested in India and conferred similar (≈80%) protection as that provided by the injectable vaccine. However, possibly because of the bile, the vaccine also occasionally caused diarrhea; this and the “difficulty and costliness of preparing oral vaccines” led the then world experts Pollitzer and Burrows in the 1950s to conclude that “the method of cholera vaccination per os has been given up entirely” [7]. 

The renewed interest in oral cholera vaccination was based on new knowledge about both the mucosal immune system and the mechanisms of immune protection in cholera. In the 1960s and early 1970s, the existence of a mucosal immune system, with secretory IgA (SIgA) as its major immunoglobulin, and being preferentially activated by mucosal rather than parenteral immunization became established. In cholera, as will be discussed further below, experimental studies demonstrated that specific antibodies to either the cell wall lipopolysaccharide (LPS) O antigen or cholera toxin, when present locally in the intestine, could effectively protect against cholera infection and disease. In both animals and human volunteers, oral immunization with cholera toxin (or in humans the cholera toxin B subunit) and killed cholera bacteria could, in contrast to parenteral immunization, effectively induce protective intestinal-mucosal SIgA antitoxin and anti-LPS antibody responses. Finally, both experimental and epidemiologic studies indicated that, in contrast to the very limited and transient immune protection conferred by parenteral cholera vaccination, convalescents from cholera disease were protected against reinfection and disease for several years. These findings provided the basis for the development of the first effective OCV, the combined killed *V. cholerae* whole-cell/cholera toxin B-subunit vaccine (Dukoral™), as well as for the subsequent OCVs that are currently available. 

## 3. Innate and Adaptive Cholera Immunity 

Individual resistance to cholera infection and disease depends on a combination of innate host factors and adaptive immunity acquired by a previous infection or vaccination. The short description below focuses on those aspects that directly guided the development and/or explains the effects of current and predicted future OCVs. A comprehensive review was recently published, which is referred to for further details and supportive references [8].

Stomach acidity and ABO blood groups are the most studied innate host factors of importance for susceptibility to cholera. A low gastric acid level is associated with an increased incidence and severity of cholera disease [9]. Likewise, individuals of blood group O are at increased risk of severe cholera due to both *V. cholerae* O1 El Tor and *V. cholerae* O139 [10,11]. It was proposed that cholera might have selected for the genetically low prevalence of blood group O in the Bengal population [10]. 

The innate immune response is upregulated in cholera. Numerous innate immune response mediators, e.g., nitric oxide, TNF-α, and IL-1β cytokines, and several defensins and other bactericidal proteins are elevated in both blood and stool during the early stage of cholera infection [12,13,14,15]. In fact, whole-genome microarray analysis of duodenal biopsies from acutely infected cholera patients indicates that the majority of upregulated genes encode for innate response proteins [13]. Notably, the two most important protective antigens in the subsequent adaptive immune response, namely, *V. cholerae* LPS and cholera toxin, are also the predominant stimulators of innate immunity in cholera infection, including the activation of the NF-κB and IL-1 systems, which are critical for promoting mucosal IgA immune responses [13,15]. 

The adaptive immune response in cholera-infected or orally immunized individuals is complex. It comprises intestinal-mucosal SIgA, as well as serum IgA, IgG, and vibriocidal antibodies, and at the cellular level, e.g., antibody-secreting cells, T cells, and of special importance for long-term protection, memory B and T cells, are involved [8]. Immune protection, both in convalescents recovering from cholera disease and after oral immunization, is mediated by locally produced intestinal-mucosal SIgA antibodies [6,16]. The primary target antigens for immune protection are the LPS O antigen and cholera toxin [6,17]. The most studied correlate of adaptive immunity to *V. cholerae* is the serum vibriocidal antibody titer. Vibriocidal antibodies, which are mainly IgM directed against the LPS O antigen, increase with age in cholera endemic areas and are then associated with a reduced risk of getting cholera disease. However, these antibodies are only a surrogate marker for the intestinal-mucosal immune status and do not directly mediate or contribute to protective immunity. For instance, parenteral vaccines confer only limited and short-lived protection, even though they induce extremely high vibriocidal antibody titers. 

### 3.1. Protective Antibodies and Mechanisms 

The protective antibacterial antibodies are mainly, if not exclusively, directed against the O1 LPS [6]. The O1 LPS has a major group-specific epitope(s) “A” shared between the Inaba and Ogawa serotypes and an additional serotype-specific “B” (Ogawa) or “C” (Inaba) epitope; only a methyl group on the B epitope distinguishes Ogawa from the epitope C of Inaba [18]. Both cross-reactive and serotype-specific anti-LPS antibodies contribute to protection [6]. However, most anti-LPS antibodies after cholera infection or vaccination are directed against the shared A epitope(s) leading to predominantly serotype cross-reactive immune protection. In particular, infection with *V. cholerae* Inaba induces strong protection against subsequent cholera episodes irrespective of serotype, whereas infection with Ogawa gives rise to more serotype-specific protection [19]. Similar to the situation for *V. cholerae* O1, antibacterial protective immunity induced by *V. cholerae* O139 infection or oral immunization is mediated predominantly by antibodies to (O139) LPS, and there appears to be no cross-protection between the 01 and 0139 serogroups. The protective significance, if any, of other antibacterial antibodies, including the antibodies against the toxin-coregulated pilus (TCP) and mucinase antigens that are known to contribute to intestinal colonization by *V. cholerae*, remains to be defined. The specific mechanism(s) whereby antibodies protect against *V. cholerae* LPS are also not fully understood. Their binding to LPS extending onto the flagellar sheath is known to inhibit bacterial motility in vitro, but other effects may also contribute in vivo, such as interference with bacterial biofilm formation or epithelial attachment. 

There is also a clear protective role for mucosal antibodies against cholera toxin. The antitoxic antibody response is mainly directed against the B subunit pentamer and protects by blocking toxin binding to target cells. Antibodies against the A subunit induced after infection may have some, but a relatively marginal, protective effect. An important observation guiding the design of the whole-cell/B subunit OCV is the synergistic protective effect of mucosal antibacterial and antitoxic antibodies [17]. While antibodies against LPS and B subunit antigens can independently protect against disease by inhibiting bacterial colonization and toxin binding, respectively, the combined protective effect is strongly synergistic (the multiple of their individual effects). 

### 3.2. The Intestinal-Mucosal Cholera Immune Response and Immunologic Memory

In contrast to the at best short-lasting protection that is seen after parenteral cholera vaccination, the protection found after cholera disease or oral vaccination has a duration of several years. American volunteers that were experimentally infected with virulent *V. cholerae* were protected when rechallenged 3 years after the first infection [20]. Likewise, epidemiologic studies in Bangladesh indicated that convalescents from a first episode of clinical cholera had a 90% reduced risk compared to controls to attract a new clinical reinfection during a 3-year follow-up period [21]. Similarly, immunization with an OCV conferred protection lasting for at least a 3-year period.

**Figure 3:**
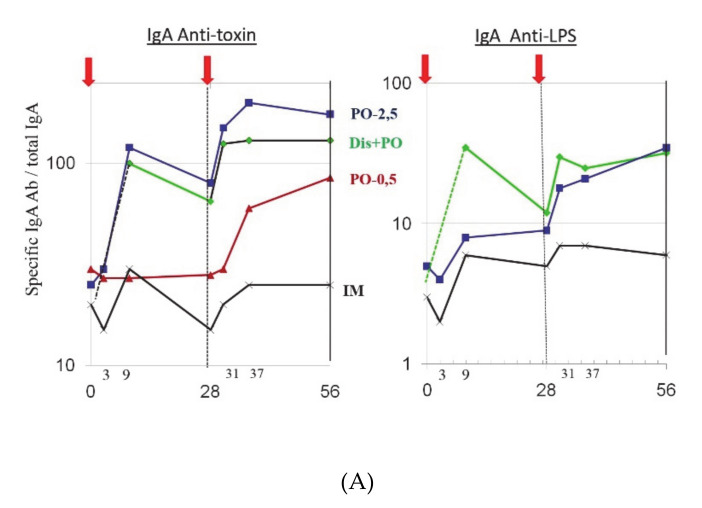

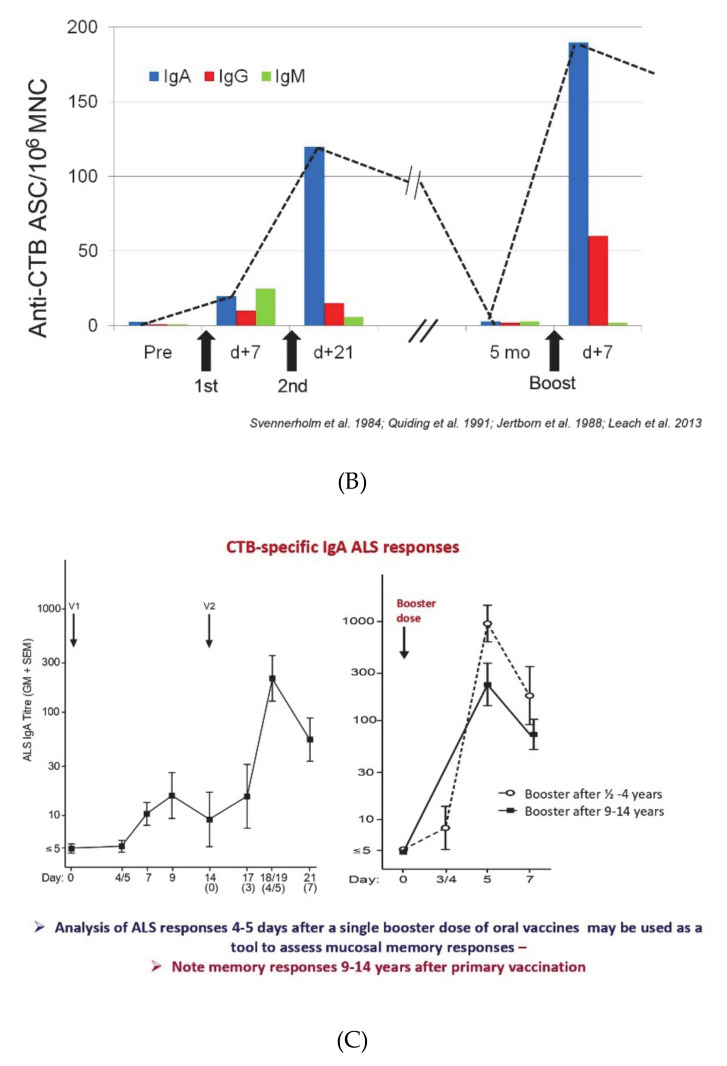
Intestinal IgA antibody responses and immunologic memory in cholera after oral immunization or infection. (**A**) IgA antibody responses to cholera toxin (B subunit) and LPS O antigen in intestinal lavage from adult Bangladeshi volunteers after cholera disease and/or immunizations with a B-subunit/whole-cell cholera vaccine that was administered orally (PO) or intramuscularly (IM) at an interval of 28 days. Two oral vaccine doses (in which the B subunit amounts were either 2.5 or 0.5 mg) induced antitoxin and anti-LPS intestinal IgA responses that were fully comparable to those measured after disease; in contrast, the IM route was ineffective. Adjusted from [16]. (**B**) Intestinal immune response kinetics in Swedish healthy adults after a first and second primary immunization with oral B subunit/whole-cell cholera vaccine (Dukoral) and after a single booster dose 5.5 months later; immune responses were examined as specific anti-B subunit antibody-secreting cells (ASC) in mononuclear cells (MNC) that were isolated from duodenal mucosal biopsies at various time points. Adapted from [22]. (**C**) Long-lasting immunologic memory to cholera B subunit found in Swedish adult volunteers for at least 9–14 years after an initial two-dose immunization regimen with the Dukoral OCV; this was demonstrated by giving a single booster dose at different times after the primary immunizations and finding intestine-derived IgA responses that were superior to those seen after a first dose in concomitantly first-time immunized volunteers and fully comparable in magnitude and kinetics to the second-dose responses in the latter individuals. Adapted from [23].

Even so, the gut mucosal SIgA anti-LPS and antitoxin responses after a cholera infection or oral vaccination are of much shorter duration; they peak after 1–2 weeks and then wane over a 4–9-month period (see Figure 3A,B [16,22]). Different from the generalized immune system, which meets only a few foreign antigens at a time and can then afford to respond vigorously to these antigens, the gut mucosal immune system is exposed to thousands of ingested antigens every day and has to economize on its response. By way of compensation, explaining the several-year-long protection that is seen after cholera disease or oral immunization, the mucosal response is associated with the development of a very long-lasting immunologic memory, which can mount a rapid anamnestic mucosal response upon renewed encounter with the antigen/pathogen even many years later. Thus, in Swedish volunteers who had received a standard two-dose initial immunization via an OCV, a rapid efficient recall intestinal SIgA response was elicited by a renewed single dose antigen exposure more than 10 years later (Figure 3C [23]).

However, it is noteworthy that immunization by the parenteral route can also elicit a SIgA response in people whose intestinal immune system is already “primed” via previous natural exposure or oral vaccination [24]. This can explain the moderate immune protection that is observed among adults but not young children in cholera-endemic areas using the old parenteral whole-cell cholera vaccines.

## 4. Currently Available WHO Prequalified OCVs

The scientific findings identifying (i) intestinal-mucosal SIgA antibodies against either or both of *V. cholerae* O1 LPS and cholera toxin mediating the immune protection against cholera [25] and (ii) oral immunization as superior to parenteral immunization for eliciting this immunity [16] directly paved the way for the development of currently used OCVs. As mentioned, the first effective OCV that was developed was the inactivated whole-cell/cholera toxin B subunit vaccine Dukoral™, which was licensed in the early 1990s [26]. This has been followed by the licensure of additional inactivated and live attenuated OCVs. The inactivated OCVs have had the greatest success in achieving licensure and international acceptance.

Three such vaccines are recommended and prequalified by WHO (meaning that they can be purchased by United Nations agencies). These are Dukoral™, Shanchol^TM^, and Euvichol^TM^/Euvichol-Plus™, where the latter two have similar and between them identical whole-cell composition as in Dukoral but lacking the B subunit component (see Table 1). Several additional OCVs, both inactivated and live-attenuated ones, have been licensed nationally but have not received WHO prequalification. The available, licensed OCVs vaccines are briefly described below; for further details and supportive references beyond what is provided in this treatise, the reader is referred to several recent comprehensive reviews [27,28,29].

Dukoral™ (Valneva, Sweden): Dukoral™ contains a mixture of formalin- and heat-killed *V. cholerae* O1 bacteria, representing both the Ogawa and Inaba serotypes and the classical and El Tor biotypes, and a recombinantly produced cholera toxin B subunit. It was internationally licensed in the early 1990s after having been shown as safe and effective in two pivotal phase III studies. The first of these trials, undertaken in 90,000 children and women in Bangladesh, showed an 85% protective efficacy against cholera during the first 4–6 months and 50–60% efficacy over a 3-year follow-up period after two or three vaccinations [30,31]. A second trial in Peruvian soldiers, all with blood group O and without any previous exposure to *V. cholerae* when given two oral doses at a 2-week interval of either Dukoral or a placebo, demonstrated 86% vaccine efficacy against a cholera epidemic 6–8 months later [32]. Several large phase IV effectiveness trials in, e.g., Mozambique and Zanzibar, later confirmed the excellent safety of this vaccine and demonstrated an 80–90% protective effectiveness of a two-dose regimen of Dukoral against cholera outbreaks occurring one or two years after vaccination. The large field trial in Bangladesh also showed that because of the extensive immunological cross-reactivity between the cholera toxin B subunit and the heat-labile toxin (LT) of *E. coli*, the whole-cell/B-subunit OCV tested there provided significant protection for 3–9 months against diarrhea caused by LT-producing ETEC bacteria; the overall protective efficacy was 67% against hospitalization due to LT ETEC and 85% against severe dehydrating disease [33]. These findings, together with observations in placebo-controlled randomized studies of 50–70% protection against LT-associated ETEC diarrhea after two doses of Dukoral in European travelers to North Africa [34] and U.S. travelers to Latin America [35], have made Dukoral widely used as a travelers’ vaccine for both cholera and ETEC diarrhea. 

Shanchol™ (Sanofi-Shantha Biotechnics, India): In the late 1980s, the technology for OCV manufacturing was transferred from Sweden to Vietnam for the local production of an oral killed whole-cell OCV. This vaccine contained the same *V. cholerae* O1 components as in Dukoral but lacked the cholera toxin B subunit in order to reduce the cost and complexity of production. A two-dose vaccination was found to give 66% protection against cholera in adults and children from one year of age. In 1992, following the emergence of O139 in India and Bangladesh, the vaccine was modified to also include killed *V. cholerae* O139 cells and it was licensed nationally, first as OrcVax™ and later after modification as mOrcvax™. More than 15 million doses of OrcVax™/mOrcVax™ OCVs were used from 1998 in Vietnam’s national cholera control program, mainly in the Mekong delta 1998–2006, where cholera was prevalent at that time.

A problem preventing WHO prequalification and thereby international use of the Vietnamese OCV was that the National Regulatory Agency (NRA) of Vietnam at that time was not WHO approved. To ensure that the reformulated vaccine could be made available internationally, the International Vaccine Institute (IVI) arranged a technology transfer from Vietnam to Shantha Biotechnics in India since India had a WHO-approved NRA. A large, cluster-randomized, placebo-controlled efficacy trial was undertaken in Kolkata and demonstrated that the two-dose oral immunization had an overall 65% protective efficacy over a 3–5 year follow-up period, although efficacy in children 1–5 years of age was seen for only 2 years [36,37]. In 2009, the vaccine was licensed in India as Shanchol™ and was WHO prequalified in 2011.

Euvichol™/Euvichol-Plus™ (Eubiologics, S. Korea): To address the increasing demand for OCVs, IVI also transferred the reformulated OCV technology to Eubiologics, Seoul, Republic of Korea. This has led to the successful production of Euvichol™ OCVs, which has an identical composition as Shanchol™. Based on studies in different countries demonstrating the “non-inferiority” of Euvichol™ in comparison with Shanchol with regard to both safety and vibriocidal antibody responses, Euvichol™ received both licensure and WHO prequalification in 2016. A new plastic tube presentation, Euvichol-Plus® Plus, providing easier storage, transportation, and administration, was WHO prequalified in 2017 and is currently the dominating OCV that is used in the global cholera vaccine stockpile [38].

## 5. Nationally Licensed but Not WHO-Prequalified OCVs

The Vietnamese mOraVax™ OCV has also been the model for a third identical OCV, Cholvax™, produced by Incepta (Dhaka, Bangladesh) and licensed in Bangladesh for use in the national cholera control program [28,29]. There are also a few other, nationally licensed OCVs on the market:

OraVacs™ (Shanghai United cell Biotechnology, China): This is a dry formulation enteric-coated capsule vaccine, which contains inactivated whole-cell (WC) *V. cholerae* O1 classical biotype or El Tor biotype and recombinant cholera B subunit, and thus has a similar composition as Dukoral™. OraVacs™ is licensed in China and the Philippines for protection against cholera and traveler’s diarrhea caused by ETEC. For initial immunization, three capsules are taken on days 0, 7, and 28 [28,29].

Vaxchora (PaxVax, United States): Different from the other licensed OCVs, this is a live-attenuated, single-dose vaccine developed by Levine et al. [39]. It consists of lyophilized V. cholerae “CVD 103-HgR” O1 bacteria that are derived from a classical Inaba strain (569B) via the deletion of the cholera toxin A subunit gene, and with its long development process since the 1980s, it has acted as a “role model” to guide essentially all the subsequent live OCVs that are currently under development (see next section). Previous efforts to generate live-attenuated cholera vaccines via random chemical mutagenesis had resulted in unacceptably reactogenic vaccine candidates. Even after modern genetic technologies were used to specifically delete the cholera toxin A subunit gene, the resulting vaccine strains continued to cause diarrhea in vaccinated volunteers, although with a much-reduced severity compared to the parent wild-type strains. It was only when this technology was applied to a *V. cholerae* strain 569B with known poor colonizing ability that an acceptably safe and yet immunogenic vaccine strain, CVD 103 HgR, could be generated. This vaccine was initially manufactured under the trade names Orochol™ and Mutacol™ (Swiss Serum and Vaccine Institute Berne), but the production was stopped for market reasons after a large field trial in Indonesia had failed to show significant efficacy [40]. Recently, the US FDA approved CVD 103-Hg under the name of Vaxchora™ for use in U.S. travelers based on human volunteer studies showing that the vaccine is well tolerated and gives up to 90% protection against a cholera challenge with either the Inaba or Ogawa *V. cholerae* O1 serotype for 3–6 months after a single dose immunization [28,29]. As of 2021, Vaxchora is also approved for use in European travelers from age 2 years.

## 6. Generation of Herd Protection Can Markedly Increase the Protective Impact of OCVs

A very important finding when analyzing the protective effectiveness of OCVs is that in addition to their specific vaccine efficacy, they can provide strong herd protection (previously often called herd immunity). Such herd protection is due to the ability of an OCV, which is linked to its vaccine-specific efficacy, to reduce the within-community transmission of *V. cholerae*, thus also protecting non-vaccinees who reside in vaccinated neighborhoods, as well as increased protection in vaccinees. The magnitude is proportional to the vaccination coverage of the target population. The generation of herd protection can markedly increase the overall protective impact of OCVs in vaccinated communities. This no doubt had a pivotal role in changing the public health perception of the value of OCVs in the global fight against cholera.

The herd protective effect of OCVs was first shown by Ali et al. [41] when re-examining the results from the large field trial of the whole-cell/B-subunit and whole-cell only OCVs that were undertaken in Bangladesh in 1985–1988. They found that the vaccines, in addition to their specific efficacy in vaccinated individuals, also conferred indirect “herd protection” to the unvaccinated individuals of the community. They found that the magnitude of herd protection was directly proportional to the vaccine coverage in the community and could be as high as 77% in the communities with the highest (>55%) vaccination coverage. Since in many settings, cholera cases occur with a marked clustering in space and time due to a frequent fecal–oral spread of cholera from an index case to other members of the same household and secondarily to neighboring households, with an up to 36-fold higher risk of secondary cholera observed among individuals living within 50 m of a confirmed cholera case in the first 3 days compared to the risk among individuals living elsewhere in the community, the herd protection effect is highest in a “ring” around a confirmed cholera case [42,43,44,45].

The ability of killed OCVs to confer herd protection has been repeatedly confirmed, e.g., in studies of Dukoral™ in Zanzibar and of Shanchol™ in India and Bangladesh. The combination of direct and indirect protection induced by OCVs significantly increases the overall protective impact of vaccination. The combined effect can be substantial even with relatively modest vaccination coverage. Thus, the findings from several large studies in different settings in Asia, Africa, and Hispaniola indicate that the available WHO prequalified OCVs with their 60–70% direct vaccine efficacy can confer almost complete elimination of cholera at vaccination coverages exceeding 50% [46,47]. This makes OCVs highly cost-effective by WHO measures, both in terms of numbers of lives saved and disease cases averted in relation to expense.

It is also important to note that the herd-protection effect of killed OCVs has been consistently found using various study designs. These have included individually randomized controlled clinical trials, cluster-randomized clinical trials, observational cohort studies, and observational case–control studies. Using all of these study designs, significant herd protection has been observed in unvaccinated persons, as well as in the community as a whole [45].

## 7. From Dismissal to Universal Acceptance of OCVs as an Important Public Health Tool for Cholera Control

As mentioned, an effective, WHO-prequalified OCV (Dukoral) has been available since the early 1990s, and was accompanied in the 1990s and 2000s with more affordable, locally produced OCVs, first from Vietnam and then from India (Shanchol). It is therefore remarkable that despite the consistent, positive results showing safety, protective efficacy, and feasibility, as well as herd protection that could further increase the overall impact, it took so long for (parts of) the WHO and the international public health community to change their negative attitude to using OCVs in cholera control programs. The positive experience from Vietnam of using an OCV in its national cholera control program since 1998 did not change the global policy.

### Why Did It Take So Long before OCVs Were Accepted for Global Control of Cholera? 

For many years, there were concerns among parts of the WHO that the introduction of OCVs might have a negative impact on the national implementation of oral rehydration therapy (ORT) and water, sanitation, and hygiene (WASH) activities. One concern was that people might feel safe by being protected by the vaccine and, thus, would be less compliant with WASH practices; another concern was that access to OCVs would take away the pressure on communities and governments to make needed investments toward sustainable access to clean water and adequate sanitation. These concerns are now seen as unfounded. Instead, WASH interventions and cholera vaccination should work well and probably even synergistically together, with OCVs improving the effectiveness of WASH and vice versa. Immunization will decrease the proportion of susceptible individuals in the community and reduce environmental contamination, thus helping to stop transmission of the disease and improving the effectiveness of WASH interventions. Conversely, WASH could make cholera vaccination more effective by reducing the risk of ingestion of a very high dose of *V. cholerae*.

The event that was pivotal for a rapid change in attitude to public health use of OCV was the cholera epidemic in Haiti in 2010. In the wake of a big earthquake, Haiti was hit by a devastating cholera epidemic, which in the first year caused >8000 cholera deaths. It soon became obvious how ineffective the traditional cholera control methods were for interrupting the spread of the epidemic. This and strong evidence that cholera had been brought to Haiti by UN peacekeeping staff from Nepal, and probably also the geographic proximity to the USA, created a rapidly increasing pressure on the WHO to use OCVs in the fight against cholera in Haiti. This rapidly led to strengthened recommendations from the WHO in 2010 to use OCVs both for the prevention of endemic cholera and interrupting and preventing the spread of cholera in outbreaks. It also led to the important decision to establish, with support from the GAVI, a global OCV stockpile for use primarily in settings afflicted by or at imminent risk for a cholera outbreak.

OCVs have been used in several settings in Haiti since 2012. It has shown excellent effectiveness, even when applied in the midst of the ongoing epidemic [46] and has also conferred long-lasting protection. A recently published study found the average 4-year effectiveness of two OCV doses to be 76% [47].

## 8. Challenges Ahead for the Elimination of Cholera

There is now great hope that the new global commitment expressed in the “Ending Cholera: A Global Road Map to 2030” program by the WHO and many partners will be revolutionary in the global war against cholera. However, there are still many challenges before its goals to reduce cholera deaths by 90% and eliminate cholera transmission in most of the currently afflicted countries will become a reality. Success will require a sustained political will at all levels, adequate sustained financing for the whole period, a motivated global health community, and an effective research and development program.

A political challenge, which is seen by many as critical for achieving the Global Roadmap’s goals, will be to convince India to use OCVs in the public health control of cholera, especially in West Bengal. Cholera is still prevalent in the West Bengal region and global genomic studies have convincingly documented that each of the major waves of the global spread of cholera during the seventh pandemic did originate from the Bay of Bengal region, i.e., the West Bengal of India and Bangladesh [48]. The same is true for each of the 10–15 introductions and reintroductions of cholera into African countries in recent years [49]. It remains unknown which environmental or human conditions fueled the Bengal incubator for the seventh pandemic *V. cholerae* lineages (and probably previous ones). However, it seems clear that the implementation of all available control methods, including the extensive use of OCVs in the populations of the Bay of Bengal, should be a priority in the global elimination efforts. The government of Bangladesh has stated it will use OCVs broadly in its national cholera control program; hopefully, India will soon do the same.

Another urgent challenge is to increase the availability of OCVs to the levels required for the implementation of the Global Roadmap. The OCV global stockpile, which was started in 2013 at a level of only 2 million doses, has now increased to 20–25 million doses annually with financial support from the GAVI. More than 50 million doses have been used in more than 100 mass vaccination campaigns in 22 countries. However, this is still significantly below the OCV requests from countries, which already exceed 50 million doses annually, mainly for outbreak control use. With the projected further use of OCVs by the Global Roadmap to be focused on prevention of endemic cholera (or cholera reintroduction) in “hotspots” the annual OCV needs will exceed 100 million doses for the coming 5–10 year period. The GAVI has recently estimated that more than 1.5 billion doses will be required globally for combatting endemic cholera in 2020–2029 [50]. Even if restricted only to the GAVI-supported countries (therefore, excluding India), the OCV needs are expected to increase to approximately 64 million doses by 2021, reaching 74 million doses in 2022, and then stabilizing at about 65–70 million doses per year from 2025 onwards. The travelers and military markets are also expected to increase from about 1.3 to 2 million doses/year over the next ten years.

Besides funding, these projected needs will require a major expansion of the global OCV production capacity, including the attraction of additional manufacturers. The latter may, in itself, be a challenge given the limited commercial market for OCV. It will be important that the GAVI, UNICEF, and other purchasers can balance the lowest-possible-cost ambition against the risk of deterring manufacturers when negotiating with existing and potential new manufacturers.

Outside the OCV supply problem, operational research is needed to define and evaluate the best ways of using OCVs together with other interventions in different settings. When a broad range of cholera experts and stakeholders recently identified the most important knowledge gaps and established a priority list of key research questions for achieving the goals of the Global Roadmap [51], the top five priorities were all focused on the best use of OCVs (Table 2).

One important question is whether (or when) the prescribed two-dose regimen of an OCV can be modified to a single-dose administration, which would be easier to deliver and, if working, would allow limited doses of OCVs to vaccinate twice as many people. The answer to this question is highly context dependent.

### 8.1. Prevention of Endemic Cholera 

Large studies in Bangladesh and high-endemicity regions have shown that, while significant protection can be achieved among adults and children above 5 years of age after a single dose of an OCV, children below 5 years of age remain largely unprotected unless they receive an initial two-dose regimen. These findings are consistent with immunological studies showing that a single-dose OCV could effectively elicit (“boost”) a protective immune response in individuals who were already immunologically “primed” by previous natural exposure to *V. cholerae*, whereas it takes two doses to induce an effective intestinal-mucosal immune response in immunologically “naïve” individuals, such as the whole population in previously unexposed communities and young children in endemic settings. 

### 8.2. Use for Outbreak Control

When OCVs are used for the control of an outbreak in a known high-endemicity hotspot and/or late in an outbreak, it may be practical and cost-effective to use a single-dose OCV in order to maximize the number of individuals that can be reached with a first dose. This should also maximize the indirect herd protection among those not receiving a vaccine in the community, which will also benefit children below 5 years of age, even though this group may not get much direct protection from the vaccine. The aim should then be to give a second dose to as many young children as is practical after 1–2 weeks or later. In contrast, for an early OCV intervention in a cholera outbreak occurring in a setting that has previously not been exposed to cholera, only a two-dose regimen with an interval of at least one week between doses is likely to work, irrespective of age.

### 8.3. Booster Doses 

Even though the available OCVs are licensed for two-dose administration with an interval of two (one to six) weeks between the doses, which is to be followed by a recommended renewed two-dose immunization at three-year intervals, it is most likely that single-dose OCV boosting at 3-year intervals would work equally well, thereby simplifying the boosting process and saving on vaccines. There is good immunological support for this recommendation. Intestinal immunologic memory was found to be effective and long-lasting, and Swedish volunteers who had received an initial two-dose immunization with Dukoral™ and then were given a single OCV boost more than 10 years later elicited a rapid, strong anamnestic mucosal IgA antibody response, which was fully comparable to that achieved by a two-dose regimen [23] (Figure 3C).

## 9. Future Cholera Vaccines

While the currently used OCVs are effective, they have a complex multicomponent composition, which adds to production costs. They also have a less-than-ideal formulation, which adds to transport and usage costs. Several new or improved OCVs are under development, as listed in Table 3 and briefly described below.

### 9.1. Simplified Compositions of Current Types of OCVs

The three WHO prequalified OCVs are as mentioned based on (the same) three *V. cholerae* O1 strains and using both formalin and heat inactivation methods; in addition, the Shanchol and Euvichol vaccines contain a formalin-killed *V. cholerae* O139 whole-cell component. The only established protective *V. cholerae* O1 bacterial antigens are the Ogawa and Inaba LPS antigens. These antigens are exposed and preserved equally well after heat or formalin inactivation, indicating that one inactivation method is enough, with formalin being the most practical for large-scale manufacturing. *V. cholerae* O139 has almost completely disappeared as a cause of cholera since the late 1990s; therefore, the *V. cholerae* O139 component in two of the current vaccines has since been an immunologically meaningless, yet cost-adding “decoration.” Based on this, it is recommended that the current whole-cell OCVs contain only formalin-killed Ogawa and Inaba bacteria from two of the current strains, preferably Cairo 50 (Classical/Ogawa) and Phil6973 (El Tor/Inaba). Efforts are underway by Eubiologics and IVI to produce and evaluate this cost-saving vaccine.

A more fundamental simplification was developed by us and further pursued in collaboration with the MSD-Wellcome Trust Hilleman Laboratories in India. A Hikojima serotype vaccine strain (El Tor biotype) stably co-expressing the Ogawa and Inaba O1 LPS antigens was generated for use as a formalin-killed single strain OCV [52,53]. The strain was constructed by introducing a partially inactivating mutation in the *wbeT* gene that is responsible for the LPS methylation differentiating the Ogawa and Inaba serotypes. This vaccine, namely, Hillchol™, was as safe and immunogenic as the comparator vaccine Shanchol when tested side-by-side in a noninferiority phase 1/phase 2 study in adults and children in Bangladesh. Bharat Biotech in India has licensed the rights to the commercialization of this easy-to-produce, low-cost Hillchol OCV, which will hopefully soon add to the global OCV supply market.

A third, recently invented, approach to simplifying and reducing the cost of vaccine production is to grow two isogenic *V. cholerae* O1 vaccine strains, one Ogawa and the other Inaba, in a mixture in the same fermentation process and then formalin-inactivating the cell mixture. Normally, one of the strains in a bacterial co-culture rapidly outgrows the companion strain, but this problem is overcome by generating and using isogenic variants with identical growth properties that only differ in that one strain expresses the *wbeT* gene resulting in serotype Ogawa and the other, by lacking this gene, has the Inaba serotype [54].

### 9.2. Thermostable, Dry Formulation Capsule OCV 

The WHO Global Task Force on Cholera Control (GTFCC) has identified a thermostable dry formulation vaccine, ideally a tablet or capsule, as a priority for further OCV development. Such a vaccine would have significant logistical advantages over current OCVs with regard to transport, storage, and deployment. We recently described such a thermostable, low-cost OCV consisting of a lyophilized mixture of formalin-inactivated *V. cholerae* O1 bacteria and rCTB formulated in an enteroprotected capsule. We initially used the previously described Hikojima/Hillchol™ strain co-expressing the Ogawa and Inaba LPS antigens as the whole-cell vaccine component for such an experimental vaccine, namely, Hillchol-B [55]. However, we have instead now changed to use the described (co-cultured and then formalin-inactivated) mixture of isogenic Ogawa and Inaba strains [54] as the preferred whole-cell component in a dry formulation whole-cell/B-subunit capsule OCV called DuoChol™. The affordable cost, practical formulation, and increased efficacy obtained by including the B subunit and also increasing the whole-cell amount/LPS O antigen content should make DuoChol™ an attractive OCV overall and an ideal vaccine for stockpiling and use in cholera outbreaks, where rapid deployment and maximal short-term efficacy are essential.

### 9.3. Live Attenuated OCVs

Several live oral attenuated OCVs are also under development. The oldest is Peru 15 (CholeraGarde), which was derived from an O1 El Tor Inaba strain isolated in Peru in 1991. Its multiple attenuating mutations include the deletion of the entire CT operon and flanking recombination sites; deletion of flagellar genes, making the strain non-motile; inactivating the *recA* gene by inserting the coding region for CTB under the control of a heat-shock promoter. The vaccine was safe and highly efficacious in a cholera challenge study of U.S. volunteers. A single-dose regimen was also found to be safe and immunogenic when tested in Bangladeshi adults, children, and infants [56,57].

Another live OCV is the Cuban El Tor Ogawa strain *638*, which is attenuated by the deletion of the CTXPhi prophage and inactivation of the hemagglutinin/protease coding sequences (*hapA*). This vaccine was well tolerated and conferred complete protection in Cuban volunteers against a challenge with a virulent strain of El Tor *V. cholerae* 01 [58,59].

A third live OCV candidate is the VA 1.4 El Tor Inaba strain generated by Indian investigators from a clinical nontoxigenic strain that naturally lacked the CTX prophage and into which the *ctxB* gene has been inserted. The vaccine was found to be safe and immunogenic when given as a single 10^9^ CFU dose to adult volunteers in India [60].

A fourth live OCV candidate strain as yet in preclinical development is IEM 108 developed in China. It is an El Tor Ogawa strain that is naturally deficient in CTXPhi and equipped with an inserted *ctxB* gene and an *rstR* gene, which blocks the reacquisition of CTXPhi [61].

Recently, yet another live attenuated vaccine strain, HaitiV, which was derived from a variant El Tor O1 Ogawa *V. cholerae* clinical isolate from the 2010 Haiti outbreak, has attracted interest by demonstrating a rapid probiotic-like protective activity, as well as more traditional longer-lasting protective immunogenicity against experimental cholera in animals [62,63]. HaitiV harbors several genetic alterations that render it avirulent and resistant to reversion. In an infant rabbit model of cholera, oral administration of HaitiV conferred protection against challenge with a lethal dose of wild-type *V*. *cholerae* within 24 h of vaccination, which is suggestive of a “probiotic”-like protection mechanism. In germ-free female mice, oral immunization with HaitiV elicited serum vibriocidal antibodies and protected their pups from lethal challenge with virulent *V. cholerae*. It remains to be studied whether these properties of HaitiV (or a Hikojima variant of this strain) will apply in humans after immunization and subsequent challenge with wild-type *V*. *cholerae* O1 and whether the rapid “probiotic” effect is specific for HaitiV or may be seen with other and possibly all live attenuated OCVs.

Despite their significant and often impressive protective efficacy against V. cholerae O1 infection and disease, even after a single dose immunization when tested in the human challenge model, live attenuated OCVs have to date met problems that have so far prevented their acceptance for use in developing countries. This is true for both the Vaxchora OCV (which, as mentioned above, has been approved for use in travelers) and for the live attenuated vaccines under development. Apart from the initial problem of residual unacceptable reactogenicity of strains even after the cholera toxin gene had been deleted, which has been overcome by developing colonization-deficient and/or multiply mutated vaccine strains, a remaining problem has been the difficulty in balancing safety against immunogenicity of the vaccine dosage in different settings. Thus, what has been both a safe and immunogenic dosage in industrialized country volunteers has often been too low dosage for inducing an adequate intestinal-mucosal immune response in developing country populations, including those living in cholera-endemic areas. For instance, a 5 × 10^8^ CFU dose of CVD103 HgR (the predecessor of Vaxchora), which was highly immunogenic in subjects in industrialized countries, resulting in greater than 90% seroconversion of a vibriocidal antibody, elicited seroconversions in only 16% of Indonesian subjects; however, a 10-fold-higher vaccine dose resulted in seroconversion in 75–87% of vaccinated Indonesians [64].

## 10. Need for Novel Pathways for Licensing New Generation OCVs

Traditionally, the licensure of a new vaccine usually requires proof of clinical protection against the targeted disease in one or more pivotal, individually randomized, placebo-controlled double-blinded clinical trials (RCTs). For current OCVs, this was the path leading to the licensure of Dukoral™ and Shanchol™.

However, when an effective and safe vaccine already exists, such RCTs may be ethically problematic, which is now the case for the new and improved OCVs under development (as well as for vaccines under development for some other diseases). This problem was discussed at a recent “Expert meeting on evaluating new generation vaccines against infectious diseases for which there are licensed vaccines that are recommended for routine use” organized by the Wellcome Trust. The comments below reflect the (as yet unpublished) main observations and recommendations from this meeting.

When there is a validated immunological correlate of protection (ICP), this can be used as the primary measure of vaccine efficacy for similar or new-generation vaccines. When an established ICP is lacking, as is the case for cholera, a “surrogate” immune response may be used for comparing the new and existing vaccines. However, this approach requires that the protective antigen(s) of the compared vaccines are defined and identical, the routes of administration of the vaccines are the same, and the vaccines are identical or similar in composition. The Euvichol™ OCV, having the same composition as the already-licensed and WHO-prequalified OCV Shanchol™, and later Euvichol-Plus™, were licensed based on human studies demonstrating the noninferior safety and the same serum vibriocidal antibody responses as for Shanchol based on the vibriocidal antibodies being specific for the targeted key protective antigen, namely, the O1 LPS.

In some instances, vaccines can be licensed based on protective efficacy demonstrated in human volunteer challenge studies. This pathway was used to license the Vaxchora live OCV as a travelers’ vaccine in U.S. adults, but it has not allowed for licensure in LMICs or children.

For vaccines against diseases for which there is a significant unmet medical need, the EMA pathway termed “conditional marketing authorization” may also be applied. This pathway typically requires the demonstration of an immunological endpoint after vaccination that is reasonably predictive of clinical benefit, substantial data on vaccine safety, and an overall favorable risk/benefit assessment for authorization.

Thus, for future cholera vaccines, both those described above and others yet to be developed, there are alternative ways to receive either full or conditional licensure based mainly on demonstrated safety and relevant noninferior “surrogate” immunogenicity to existing OCVs. However, in these cases, there will probably be requirements for vaccine effectiveness studies post-licensure to demonstrate vaccine protection. The operational criteria for the design, conduct, analysis, and reporting of such post-licensure studies should be defined in a dialogue between the cholera scientific community and vaccine regulators.

## 11. Concluding Remarks

For those of us who have been “OCV musketeers” since the 1990s, it is of course gratifying that the WHO now describes the use of OCVs as “a game-changer in the fight against cholera” in its strategy for the global control of cholera “Ending Cholera: A Global Roadmap to 2030.” It underlines that by providing effective protection and being available now, OCVs can effectively bridge the time and resource gap until adequate WASH infrastructure may be universally available as a means for permanent prevention.

The development of OCVs is a good example of how basic research can be translated into a life-saving medical product. At the same time, the OCV story also illustrates how long and tedious the process even from an available product to public health use can be, especially for vaccines and drugs that are targeted against diseases for which there is no or minimal commercial market in higher-income countries. Cholera is a typical “disease of poverty” that mainly affects the poorest populations in low- or at best middle-income countries. Had it not been for the fact that Dukoral™, the first effective OCV, in addition to protecting against cholera, also protects against diarrhea caused by ETEC through its B subunit component and, thus, offered a commercial market for Dukoral as a traveler’s vaccine, the licensure of an effective cholera vaccine would almost certainly have been delayed by many years.

Several challenges, both financial and political, remain to accomplish the set goals of the Global Roadmap to have decreased cholera deaths by at least 90% and largely eliminated cholera transmission by 2030. Yet, it is gratifying that the earlier question regarding whether to use OCVs has now changed to ”When, how and where should OCV be used most effectively to control endemic cholera and interrupt cholera outbreaks?” and “How can we increase manufacturing of existing and new OCVs to meet current and future needs?”

## Figures and Tables

**Table 1:** Composition of WHO prequalified inactivated OCVs.

Dukoral™	Shanchol™, Euvichol™	Quantity per Dose
*V. cholerae* O1 Inaba classical strain Cairo 48 Heat inactivated	Same as in Dukoral	300 EU* LPS (~2.5 × 10^10^ bacteria)
*V. cholerae* O1 Ogawa classical strain Cairo 50 Heat inactivated	Same as in Dukoral	300 EU of LPS (~2.5 × 10^10^ bacteria)
*V. cholerae* O1 Ogawa classical strain Cairo 50 Formalin inactivated	Same as in Dukoral	300 EU of LPS (~2.5 × 10^10^ bacteria)
*V. cholerae* O1 Inaba E1 Tor strain Phil 6973 Formalin inactivated	Same as in Dukoral	600 EU of LPS (~5 × 10^10^ bacteria)
—	*V. cholerae* O139 strain 4260B Formalin inactivated	600 EU of LPS (~5 × 10^10^ bacteria)
Cholera toxin B subunit (rCTB)	—	1 mg

*EU stands for ELISA units, referring to the capacity of the vaccine to bind specific anti-LPS antibody in an internationally used Inhibition-ELISA method for quantification of LPS antigen.

**Table 2:** Top Five Priorities of the Cholera Roadmap Research Agenda of January 2021 (from [51]).

1	What are the optimal oral cholera vaccine schedules (number of doses and dosing intervals) to enhance immune response and clinical effectiveness in children that are 1 to 5 years of age?
2	What are potential delivery strategies that can be used to optimize oral cholera vaccine coverage in hard-to-reach populations (including during humanitarian emergencies and areas of insecurity)?
3	Is there additional benefit to adding WASH packages, for example, household WASH kits, to an oral cholera vaccine campaign?
4	What is the optimal number of doses of oral cholera vaccine to be used for follow-up campaigns in communities previously that were vaccinated with a two-dose schedule?
5	Can the impact of an oral cholera vaccine on disease transmission, morbidity, and mortality be maximized by targeting specific populations and/or targeted delivery strategies?

**Table 3:** Not yet licensed oral cholera vaccines under development.

Type of Vaccine	Name/Description/Stage of Development
Simplified compositions of current OCVs	Formalin-killed Cairo 50 (Classical/Ogawa) and Phil6973 (El Tor/Inaba), which is in preclinical development in South Korea. Hillchol™, which contains formalin-killed Hikojima El Tor strain MS1568 and is developed in India and Sweden. This OCV is in planned phase 3 testing in India. Formalin-killed co-cultured isogenic El Tor Ogawa and Inaba, which is in preclinical development in Sweden.
Thermostable dry formulation capsule OCV	DuoChol™, which contains lyophilized formalin-killed isogenic El Tor Ogawa and Inaba strains and recombinant cholera toxin B subunit in an enteroprotected capsule. This OCV is in preclinical development in Sweden.
Live attenuated OCVs	Genetically engineered *V. cholerae* O1 strains with deletions of ctx and other mutations: Peru 15, which is derived from an O1 El Tor Inaba clinical isolate from 1991 in Peru, and is developed in the USA. This OCV has displayed phase 1 (also including challenge) and phase 2 immunogenicity. El Tor Ogawa strain *638,* which is developed in Cuba. This OCV has displayed phase 1 (also including challenge) and phase 2 immunogenicity. VA 1.4 El Tor Inaba strain, which is developed in India. This OCV has displayed phase 1 immunogenicity. IEM 108 El Tor Ogawa strain, which is developed in China. This OCV has displayed phase 1 immunogenicity. HaitiV, which is derived from a variant El Tor O1 Ogawa isolated in Haiti, and is in preclinical development in the USA.
